# Resequencing of *Capsicum annuum* parental lines (YCM334 and Taean) for the genetic analysis of bacterial wilt resistance

**DOI:** 10.1186/s12870-016-0931-0

**Published:** 2016-10-28

**Authors:** Yang Jae Kang, Yul-Kyun Ahn, Ki-Taek Kim, Tae-Hwan Jun

**Affiliations:** 1 Plant Systems Biology, School of Life Sciences Weihenstephan, Technical University of Munich, Freising, Germany; 2 Vegetable Research Division, National Institute of Horticultural & Herbal Science, Rural Development Administration, Wanju-gun, Republic of Korea; 3 The Foundation of Agricultural Technology Commercialization and Transfer, 441‑100 Suwon, Republic of Korea; 4 Department of Plant Bioscience, Pusan National University, Miryang, Republic of Korea

**Keywords:** Pepper, Bacterial wilt, Resequencing, SNP, YCM334, Taean

## Abstract

**Background:**

Bacterial wilt (BW) is a widespread plant disease that affects a broad range of dicot and monocot hosts and is particularly harmful for solanaceous plants, such as pepper, tomato, and eggplant. The pathogen responsible for BW is the soil-borne bacterium, *Ralstonia solanacearum*, which can adapt to diverse temperature conditions and is found in climates ranging from tropical to temperate. Resistance to BW has been detected in some pepper plant lines; however, the genomic loci and alleles that mediate this are poorly studied in this species.

**Results:**

We resequenced the pepper cultivars YCM344 and Taean, which are parental recombinant inbred lines (RIL) that display differential resistance phenotypes against BW, with YCM344 being highly resistant to infection with this pathogen. We identified novel single nucleotide polymorphisms (SNPs) and insertions/deletions (Indels) that are only present in both parental lines, as compared to the reference genome and further determined variations that distinguish these two cultivars from one another. We then identified potentially informative SNPs that were found in genes related to those that have been previously associated with disease resistance, such as the R genes and stress response genes. Moreover, via comparative analysis, we identified SNPs located in genomic regions that have homology to known resistance genes in the tomato genomes.

**Conclusions:**

From our SNP profiling in both parental lines, we could identify SNPs that are potentially responsible for BW resistance, and practically, these may be used as markers for assisted breeding schemes using these populations. We predict that our analyses will be valuable for both better understanding the YCM334/Taean-derived populations, as well as for enhancing our knowledge of critical SNPs present in the pepper genome.

**Electronic supplementary material:**

The online version of this article (doi:10.1186/s12870-016-0931-0) contains supplementary material, which is available to authorized users.

## Background

Bacterial wilt (BW) is a common plant disease that affects a wide array of diverse hosts, ranging from dicots to monocots. It is especially harmful for a number of solanaceous crops, including peppers, tomatoes, and eggplants. BW is caused by the bacterial pathogen, *Ralstonia solanacearum,* which can adapt to diverse temperature conditions and is commonly found in soil from a broad distribution of tropical to temperate climate regions [1]. *R. solanacearum* infects plants through cracks, such as wounds, root tips, and lateral root emergence sites, and eventually colonizes the root cortex. After invading the xylem vessels transporting water and soluble mineral nutrients from root throughout the plant, the bacterial pathogen can rapidly multiply, filling up and blocking the xylem. Eventually, infection with *R. solanacearum* leads to host wilting and quickly results in plant death. Because of these destructive symptoms, this bacterium is ranked second out of the top 10 pathogens that have importance with regards to economic and scientific consequences [2].

In tomato, the genomic regions that confer resistance against BW have been characterized; the Bwr-12 region is known to confer strong resistance against BW and is specific to phylotype I (Asian) strains. The Bwr-6 region confers weaker resistance than Bwr-12, and this quantitative traits loci (QTL) is specific to both phylotype I and II strains [3, 4]. The Bwr-6 region in particular is also known to mediate broader resistance against other diseases, including root-knot nematodes, potato aphids, *Cladosporium fulvum*, *Oidium lycopersicon,* Tomato yellow leaf curl virus, and Alfalfa mosaic virus [5]. Resistance to BW has also been detected in pepper plants; however, the genomic loci and alleles that mediate resistance responses against BW are poorly understood in this species.

Pepper (*Capsicum annuum*) belongs to Solanaceae family and is one of the most prevalent and economically important crops in the world. The pepper genome has 12 chromosomes and is estimated to be 3.48 Gb [6]. As is the case for other solanaceous species, *R. solanacearum* has been isolated from wilting field-grown pepper in south Florida and has also been observed in Japan [7, 8]. Considering the wide host range and adaptability of *R. solanacearum*, we predict it will be necessary to utilize the collection of the pepper germplasms resistant to BW, in order to breed elite cultivars that can counteract the destructive effects of this disease. However, there have been few efforts to select resistant donor accessions from the pepper germplasm collection, and the biological knowledge required to carry out molecular breeding in this population is limited.

Next generation sequencing (NGS) technologies have significantly advanced genomic studies, enhancing both the amount and accuracy of sequencing data that can be affordably obtained. These techniques have almost completely replaced laborious and time-consuming gel-based genotyping procedures, at least for marker development, and consequently, the majority of beneficial crop species have been sequenced and assembled into draft reference genomes, after which, the genomic resources for a given crop species are often enriched using resequencing strategies [9]. For example, after completion of the pepper (*C. annuum*) reference genome, which covers 87.9 % of the estimated genome size, two pepper cultivars (Perennial and Dempsey) and a wild species of pepper (*C. chinense* PI159236) were resequenced, revealing millions of single nucleotide polymorphisms (SNPs) that may discriminate between cultivars or between species [6]. Moreover, 18 accessions of pepper cultivar and two semi-wild accessions were resequenced to investigate how artificial selection traces present in the pepper genome correlate with pepper breeding history [10]. Parental lines of breeding populations were also resequenced to identify the causal regions conferring resistance against the Potato virus Y [11]. The availability of NGS technology and the *C. annuum* reference sequence provides us with the opportunity to employ this resequencing strategy to address more specific and practical questions in genome-assisted breeding schemes to cope with BW.

Here, we resequenced *C. annuum* YCM344 and Taean, which are parental recombinant inbred lines (RIL) that are distinguished by differing resistance against BW. Compared to the previously known SNPs, we identified novel variations existing only in both parental lines, as well as those that distinguish these cultivars from one another. We further annotated informative SNPs by identifying those variants found in genes related to known disease resistance genes, such as the R genes and stress response genes. Moreover, via comparative analysis, we identified SNPs located in genomic regions that are homologous to known BW resistance genes in the tomato genomes. Using these SNP profiling data, we can narrow down the list of informative SNPs to identify those likely to be involved in BW resistance in pepper, and they can be practically used for marker-assisted breeding schemes with these populations.

## Results

### Whole genome resequencing of parental lines for BW resistance breeding

**Fig. 1:**
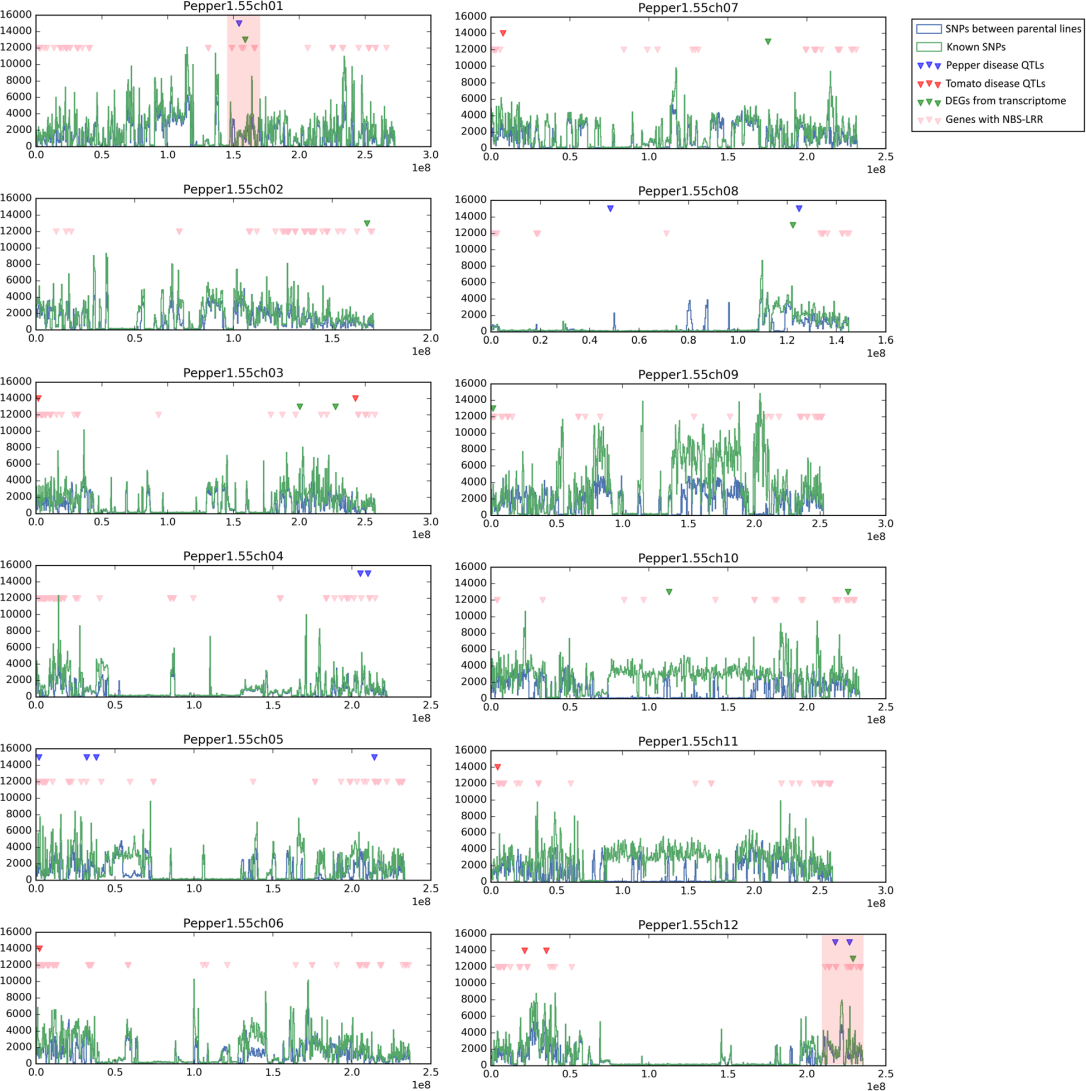
Genomic distributions of genetic markers and candidate genes in pepper genome. Blue and green lines show histogram of SNPs between parents and known SNPs, respectively. Blue and red inverted triangles point known disease-resistance QTL from pepper and tomato with non-Syn SNPs (Additional file 1: Table S3, Table 3), respectively. Green and pink inverted triangles indicate differentially expressed genes with non-Syn SNPs (Table 4) and NBS-LRR genes, respectively

The parental lines, YCM334 and Taean, were selected based on their differing resistance to BW disease; YCM334 displays high levels of resistance and Taean is susceptible. These were resequenced using the Illumina Hiseq2000 platform, producing reads totalling 36.88 Gb and 35.95 Gb, respectively, which provide approximately 10× coverage of the pepper genome (estimated size of 3.48 Gb) (Additional file 1: Table S1) [6]. For the precise call of sequence variations, we trimmed the reads based on quality, using the SolexaQA package (Additional file 1: Table S1) [12]. The processed reads from both YCM334 and Taean were then successfully mapped to the pepper reference genome sequence (version 1.55) with mapping rates of 93.56 % and 93.55 %, respectively. Using the SAMtools software package [13], we identified genomic variations between the reference genome and each cultivar, including SNPs and insertions/deletions (Indels). A total of 7,002,670 and 6,779,745 SNPs were found, with frequencies of 2.01 SNPs/kb and 1.95 SNPs/kb for YCM334 and Taean, respectively (Fig. 1 and Table 1). Of these, around 95 % were identified as being homozygous, suggesting our well-developed inbred lines can be used as parental lines for a breeding population, for example, to produce RILs.

**Table 1:** Summary of SNPs from YCM334 and Taean against reference genome

Cultivar	Region	Number of SNPs				Number of Indels			
		Number of total SNP	Homozygous SNP	Heterozygous SNP	Ambiguous SNP^a^	Number of total Indel	Homozygous Indel	Heterozygous Indel	Ambiguous Indel
YCM334	intergenic-region	6,885,942	6,592,983	109,558	183,401	347,901	172,360	25,321	150,220
	genic-region	116,728	106,531	3,735	6,462	11,199	4,788	1,071	5,340
	coding sequence (CDS)	42,786	36,950	2,146	3,690	1,918	683	224	1,011
	Intron	73,942	69,581	1,589	2,772	9,281	4,105	847	4,329
	Total	7,002,670	6,699,514	113,293	194,563	359,100	177,148	26,392	155,560
Taean	intergenic-region	6,659,677	6,344,381	120,733	120,733	329,910	160,997	24,753	144,160
	genic-region	120,068	109,622	3,797	6,649	11,320	4,878	1,014	5,428
	coding sequence (CDS)	44,392	38,238	2,186	3,968	1,880	698	202	980
	Intron	75,676	71,384	1,611	2,681	9,440	4,180	812	4,448
	Total	6,779,745	6,454,003	124,530	127,382	341,230	165,875	25,767	149,588

^a^SNPs that have not enough depth coverage to determine whether home/hetero they are

When compared to the previous resequencing efforts of Kim et al. [6]*,* which found that the pepper cultivars Perennial and Dempsey contain 10.9 and 11.9 million SNPs, respectively, our resequencing effort revealed an additional 2,748,164 SNPs that are specific for our parental lines (Additional file 1: Table S2). Further, a total 177,148 and 165,875 homologous Indels were identified in YCM334 and Taean, respectively, as compared to the reference genome. Of these, 683 and 698, respectively, were located within the coding sequence (CDS). The reliability of SNP calling was confirmed using the Sanger sequencing method on gene CA04g03400 (Additional file 1: Figure S1). This result suggests that there were no false positive SNP callings, although two false negatives were found on chromosome 4:10285038 [T/A] and 10285121 [A/G]. Analysis with the Bowtie2 [14] SNP calling pipeline decreased the false negatives, while also adding false positives (Additional file 1: Figure S1). Hence, we selected conservative BWA-based pipeline to have more confident genotypes for further analysis.

### Comparison between YCM334 and Taean

To identify the most informative alleles, in regards to BW resistance, present in YCM334 and Taean, we compared the genotypes to one another and identified the variations. A total of 5,681,208 SNPs were found that differ between the two cultivars (Fig. 1), and based on the Indel calls from the resequencing results, we found 149,223 polymorphic Indels differing between them as well. We then designed 678,998 high-resolution melting analysis primers for high-throughput genotyping and identified 12,062 possible Cleaved Amplified Polymorphic Sequences (CAPS) marker sites in 5647 genes, based on the polymorphic information (Additional files 2 and 3). Based on the Indels, an additional six possible CAPS marker sites were identified (Additional file 4). These genetic markers can be applied for high-throughput genotyping on the breeding populations to map segregating traits, such as BW tolerance.

We further analysed the SNPs present within gene regions, which may mediate functional variations. Among the polymorphic SNPs, 106,585 were present within gene regions, and 36,678 of these were in the CDS region. Among the CDS SNPs, 23,396 showed non-synonymous (non-Syn) protein changes in 9102 genes (Additional file 5). We then identified the top 10 genes that are highly polymorphic between two cultivars based on the non-Syn SNPs (Table 2). Interestingly, the most polymorphic gene, with 39 non-Syn SNPs, was CA10g15480, which is annotated as a “Putative disease resistance protein”. This gene was assigned to the “Late blight resistance protein R1” gene family (IPR021929) by Interproscan [15]. Additionally, CA12g20430, a highly polymorphic gene with 29 non-Syn SNP, was characterized as belonging to the “Late blight resistance protein R1” gene family as well, suggesting that polymorphism of this gene family can be important for the different disease responses in two cultivars (Additional file 1: Figure S2). Other genes with a large number of non-Syn SNPs include, polyprotein, LRR like receptor kinase, N-like protein, CC-NBS-LRR, and putative phosphatidylinositol 4-kinase. These were particularly prevalent in the nucleotide-binding site-leucine-rich repeat (NBS-LRR) regions that are well-known in R genes to provide resistance against pathogens [16]. A total of 286 NBS-LRR genes showed non-Syn SNP changes between the two cultivars (Additional file 6).

**Table 2:** Top 10 genes that are highly polymorphic between YCM334 and Taean by non-syn SNPs

Genename	Annotation	Genic SNP	CDS SNP	Non-syn SNP
CA10g15480	Putative disease resistance protein	46	46	39
CA02g09500	Polyprotein	39	39	34
CA06g11250	Detected protein of unknown function	55	44	33
CA04g02500	Detected protein of unknown function	69	44	30
CA12g20430	Putative disease resistance protein	36	34	29
CA05g02730	PREDICTED: probable LRR receptor-like serine/threonine-protein kinase At3g47570-like [*Solanum tuberosum*]	75	43	26
CA12g02380	N-like protein	163	28	24
CA04g00830	Phosphatidylinositol 4-kinase, putative	195	38	24
CA03g04340	Putative CC-NBS-LRR resistance protein	30	29	24
CA12g02650	PREDICTED: probable LRR receptor-like serine/threonine-protein kinase At4g36180-like [*Solanum lycopersicum*]	93	31	23

### SNP annotation utilizing homologous pepper and tomato genes involved in pathogen resistance

The numerous high-quality SNPs that were identified using NGS can be utilized to better understand the genomic variation between two cultivars. However, it would also be informative to have annotation for certain SNPs that have possible linkage or overlap to known loci of interest. Therefore, to assign annotation for the SNP, we surveyed the literature for previous knowledge of gene function, particularly disease-related, QTL, or trait-associated markers. For this purpose, we first utilized the well-studied related species, tomato (*Solanum lycopersicum*), which is model plant from the Solanaceae family. We found tomato genes that have been associated with several bacterial, fungal, nematode, and virus diseases, and then compiled a list of the pepper genes that are highly homologous to those genes. Among them, a total of seven genes showed non-Syn changes between YCM334 and Taean, which may result in functional differences between the cultivars (Table 3). These seven genes represent strong candidate loci that in YCM334 are likely to contribute to the resistance phenotype against BW disease.

**Table 3:** Non-syn SNPs in the homologs between pepper and tomato genes where disease related QTLs have been mapped

Disease	Gene list	Donor species	tomato ID	Pubmed ID	Top hit to Pepper (based on blast score)	SNP context
Bacterial speck	Prf	*S. pimpinellifolium*	Solyc05g013280.2	11952131	CA11g02030	ATGTCAAGGGTTATAGACCC(T/G)CTTGGTATTACATGTTGTAT
						CCTCTTGGTATTACATGTTG(T/C)ATCTCTCTGATGTTGAGAAA
						TCTCATCCACTCTGGTACAA(A/C)ATTCTTTGGATTTCTGAAGT
						GCATTAGGCTATTCAGAGAA(T/A)GTGAAGGGACGGTGTGTTCT
						TCAAATACTTAGAATTGGAC(A/G)ACCTCAATATTTCACAGTGG
Bacterial spot	Bs4	*S. pennellii*	Solyc05g007850.1	14675431	CA12g06200	TGAAAATTGGTATGTAGGTG(C/A)TAACTTCTTGGGATTTTCTG
						TATTTTTCGGAAGAATTGAA(G/C)GAGTTTGGACTTCGTTTGTT
						TGTATAAAGATGAACCAACA(G/A)AACATGATGATGAAGTCCGT
Alternaria stem canker	Asc-1	*S. lycopersicum*	Solyc03g114600.2	10781105	CA03g29040	ATGCTAGGCATTGGCTAAGC(G/T)AATGATTTTTGGAGAGAAGG
						AAGAGTCGGCATGGAAGTTT(G/A)TGTACTTTCTATCTGCTGAG
Leaf mold	Cf-4, Cf-9B(Hcr9-9B)	*S. habrochaites, S. pimpinellifolium*	Solyc01g006550.2	9413991	CA12g07610	ATCCCATGAACAGCAATCCG(C/T)GCTCCTATTCCACGAAAGAG
						TGGTGAATCTTCTTCTTCTT(C/T)TTCTTCGTCCAGCTCAACTG
Fungal disease	LeEIX1, LeEIX2	*S. lycopersicum*	Solyc07g008620.1, Solyc07g008630.1	15155877	CA07g01930	AAGGCCTCTTTTGAACTCAA(C/T)AAGGGCAGCTCTCTCCTTTT
						TTCTTTTTTATCTTCTTCAT(A/C)ACCCCATGTTGATAAACGAC
						GATGGGAACTCCTCCTCCTC(C/A)TCATCCTCATCATCATCATC
						AAGAATCCCCAAAATGCGAC(C/G)AAGAAACCTAGCACCATCGA
Tobacco mosaic virus	Tm-2a, Tm-2	*S. peruvianum*	Solyc09g018220.1	17246482, 16172136	CA03g00810	CTTAAGGCAACAACAGATTG(C/A)GCCTTTGCACTTGTTGGATT
						TGTTCCCAAATATATTCGGG(T/G)TAGTGACTCTTTGATTAAAA
						AAAAGAACCCAAAATATTCT(C/A)TATGCAATCCGTAATGAAGA
						TGTTTGGGCAAAATTGGCTT(A/C)TATTAGAAAAGAACCCAAAA
						TATTTGCTCTTTCGTGCGAC(C/A)GGAAAAATAGAGCTTCAGAA
						AGACGACTATCTATCTTAAG(C/A)TCGACAAGAGTAAGCTTGAC
Nematode (root knot)	Mi1.2	*S. peruvianum*	Solyc06g008450.2	9707547	CA06g00990	TTAACCAAGTTACCGGCTCG(G/A)ATTTGAAGTTCAGTGAGGAT

To further annotate our SNPs, we also took advantage of a previous transcriptome analysis of resistance and susceptible pepper lines, which identified differentially expressed genes (DEGs) in these cultivars using *Arabidopsis* gene chip analysis [17]. The corresponding direct orthologs with the *Arabidopsis* gene ID were regarded as candidate DEGs in pepper gene model, and we identified those with non-Syn SNPs between YCM334 and Taean (Table 4). One of these, beta-galactosidase 4 (CA03g17620) contained 15 SNPs resulting in non-synonymous protein changes, and the CC-NBS-LRR family gene (CA12g19770) contained seven non-synonymous SNPs.

**Table 4:** Non-syn SNPs in the homologs between pepper and tomato genes that are reported at differentially expressed genes (DEG)

*A.th.* Gene	Description	PLAZA^a^ tomato Ortholog	Blast top hit btw. tomato and pepper	SNP context
S response				
AT5G59120	subtilase 4.13	SL09G009750	CA09g00620	TAGACCATACAAGCTAGTGC(C/T)CGGGACGGGTATCCCACTGA
		SL01G091930	CA01g19460	TACTTTCAATCTTCTTTTAG(T/C)TGTTGCAGGCAAACCAATAA
				ATTAGTACCTTGAACTCTCT(T/C)GAGTATCTGCGCTCTTGGCT
AT5G56870	beta-galactosidase 4	SL03G019890	CA03g23820	CTTGTCATTTTTATCATCCA(A/T)ATTGGCAAGAAAGGCAGCAC
				ACAATTTCATGTTAAAGTTT(A/G)CGCTGGACATTTCTAACTCA
		SL07G042220	CA02g28670	CAGAAGATTGACCCCTTCCT(T/A)GAATAATAGTGTCATCAATC
				ATTGCATGACAATTCCATTG(C/T)TTTGTTCTCATAAGCACTTC
		SL09G092160	CA03g17620	ACTCGCCGGCGATGATCGTC(A/C)AGTGTATACTTAACGCCGTT
				CTTTGCGAACTTGACAATAT(C/A)ATATCTTCCTTCAAAGTTAT
				TTTCAGCAGCCCATTTCATA(T/C)ATATCTTCCCCTTGGGACCG
				ATCATAATCATAGCTAGTGA(T/G)TTGAGCTGGGCCTCCAGCAG
				ATAACTGACCTCTTGTTTTG(G/T)TCCCAGTTTAATATACTGAG
				TTTAATTGAAGTTTGTGCGG(T/C)CACCTGTTTAAGGAATGGAA
				GATTGTGAAATACTTTCGAG(A/C)TTGCTTTTAGTGCTTAATTG
				CAAAATCGCGCATGCTATCA(A/C)TATCAATCGTTGGACTAACA
				TGATCCATTTGCCTTTCACA(C/A)TACCTTCATTTGCAACAGAC
				ATCCAGGGCAACGGGATCTG(T/C)TCTGCCTGGGGCATCAAACT
				TGACCTTTTCCCATGCTACT(T/A)AAATCCAGGGCAACGGGATC
				TGCAACCAAAGTCCAATATC(T/A)TCCTATATGGTGACCATTAA
				AGTTTGTCCTACATTTATCA(A/G)AGCCGTAAGCACCACGATAA
				TGCTTTATCCGTCAGAGAAA(T/G)TTTCCCGTCGAACTCTGAGT
				CTTGACAATGTGTCGACATG(C/G)ATCTCCAACTACGGCATTGG
AT1G53350	Disease resistance protein (CC-NBS-LRR class) family	SL12G096920	CA12g19770	AACAGATTAACAAGATGAGG(A/G)ATGACGAGCTCGTTAAGGCA
				GCGTCCTGGCTTTAAGTTAT(G/T)ATGATTTACCGTATCAGCTT
				GTGTTTTCTGTACTTGGGCA(G/A)CTTTCCGGAGGGTGAAAAGA
				GGGCTGCTGAAGAAATTATA(G/C)CATTGGAAGGTAACCAAGGA
				ATGGTTCAGGTGCAACTAGA(C/G)GAAACAATCGGAAGGATCAA
				CCTGGAGGGTCGAGACAGGC(A/G)CCATGCCTAATCTAGTTCAT
				CCAAGATTAAATCCAGAATG(T/A)TATTTTCAGGTACTCAGTCA
AT5G20080	FAD/NAD(P)-binding oxidoreductase	SL05G018520	CA10g18310	TAAAATAAATGGGGCTGACG(G/A)CCAATATCGTTCATCACCCA
				ATACGAGCAAGAGCAATATC(T/C)ATATCATTCTTAAGTCGGGT
AT3G61220	NAD(P)-binding Rossmann-fold superfamily protein	SL01G094220	CA08g06650	ACAATGCAGGAGTTGGTGGA(G/T)TCACTGCAGATGCTGATGCC
R response				
AT2G38540	lipid transfer protein 1	SL10G075100	CA10g08470	AGGGAGAGCAGCAGCTTTGC(C/G)CATGTCAATGCCCTTGATTG
		SL07G049280	CA07g10490	AATTACTCCTACAATGTAAT(C/T)TGTAACACTTTTAAGTGTTT
				ATTCTTTAGAGCCCACTCTT(T/C)TCTCAAGGAAGCAAATTTTT

^a^[22]

We also surveyed the pepper disease resistance QTLs, including those involved in resistance to *Phytophthora capsici, Colletotrichum acutatum*, and *Ralstonia solanacearum* (Additional file 1: Table S3). We found 11 reported genetic markers from the literature and a Korean patent (http://patent.ndsl.kr/). We then identified two SNP regions that are proximal to pepper disease QTL, as well as to DEGs, NBS-LRR clusters (Fig. 1). These highly overlapping regions with several annotations would also be candidate regions for mediating BW resistance.

## Discussion

The selection of parental lines with specific characteristics is critical for effective crop breeding schemes, which are highly dependent on phenotypic selection after the development of breeding populations. Once the parental lines are determined based on a target phenotype, genotypic features are also informative for developing polymorphic molecular markers that distinguish between target parental lines and can be used to trace down loci responsible for observed genetic variation. The trait mapping resolution increases along with the number of molecular markers that are applied to genotyping; however, this also increases the cost of genotyping. With the development of NGS resequencing technology, we can identify all possible polymorphisms between target parental lines and select highly informative variations based on previous knowledge, such as known gene function and QTL of the corresponding species. We can also take advantage of related model species using comparative genomics approaches [9]. These technological and analytical advances can, in fact, reduce the number of molecular markers required for genotyping and increase the efficiency of marker-assisted breeding schemes, by allowing us to assign priority on each possible molecular marker. In wheat, for example, selected molecular markers that are tightly linked to phenotype were reliably genotyped in a cost effective and high-throughput manner by a multiplexing amplicon NGS sequencing strategy [18].

In this study, we resequenced the parental lines YCM334 and Taean that display distinct BW resistance phenotypes. Our data allowed us to develop genetic markers covering the whole pepper genome that are highly informative for quantitative trait loci mapping of BW disease resistance and may be utilized in a breeding scheme to develop a resistant elite cultivar. We identified the genetic variations differing between these two cultivars and further annotated them based on previous functional knowledge, both in pepper, as well as in the related model crop, tomato. We further took advantage of the gene annotation of loci in the NBS-LRR, which are known to have disease-related functions. Although we could not determine which variations from the analysis are clearly responsible to our target trait, the SNPs and Indels identified in this study, as well as their annotation-based priority, will be valuable for genotyping RILs and near isogenic lines originating from a combination of YCM334 and Taean. Further, the overlap between the variations and our previous knowledge of their likely function also provide evidence that this breeding combination contains allele resources that would show segregation on our target trait. With 169 RILs from a cross between the parent lines, a single factor ANOVA test on quantitative resistance responses of groups classified by the genotype of one selected candidate gene showed significance (*P* < 0.05) (Additional file 7). Furthermore, the parental genotype information would be highly useful for the genotype imputation and curation for ambiguous or missing data, especially from low-coverage resequencing or genotype by sequencing (GBS) data from large numbers of individuals from breeding population, allowing us impute missing alleles in linkage with known alleles for the majority of genomic regions [19]. Thus, we predict that our analyses will be valuable, not only for the fundamental analysis of YCM334/Taean-derived populations, but also for enhancing our general knowledge of variation in the pepper genome.

## Conclusions

Resequencing of the parental lines, YCM334 and Taean, has allowed for the identification of genetic markers, such as SNPs and Indels, which distinguish these cultivars, both from the reference genome and from one another. The downstream analyses of these variations, focusing on those in gene coding regions, and comparing to previously identified genomic regions responsible for resistance, such as QTL, and functional markers, has allowed us to generate a list of highly informative genetic markers that can facilitate genetic analysis using high generation populations, such as RIL. Our results are likely to provide a valuable resource, not only for the study of pepper BW, but also for the other pepper diseases against which YCM334 displays resistance.

## Methods

### Plant materials

The *C. annuum* YCM334 and Taean germplasms were provided by the National Institute of Horticultural & Herbal Science, Rural Development Administration, in the Republic of Korea. YCM334 was originally collected from AVRDC (World Vegetable Center) and is a recombinant inbred line derived from a cross between cv. Yolo Wonder and CM334. According to our observation, YCM334 showed resistance against *R. solanacearum* and is also known to have high resistance against *P. capsici* infection, whereas Taean is susceptible to *R. solanacearum* [20].

### Analysis of NGS results

The raw sequences produced from the Illumina Hiseq2000 were processed by the SolexaQA package [12], and low-quality bases with a phred score <20 were removed using DynamicTrim which is part of SolexaQA package. After trimming, read lengths below 25 bp were removed by LengthSort function of the package prior to mapping analysis. The processed reads were then mapped to the reference sequences using BWA software [21] with the following options: maximum number of gap extensions (−e) = 50, seed length (−l) = 30, maximum differences in the seed (−k) = 1, number of threads (−t) = 16, mismatch penalty (−M) = 6, gap open penalty (−O) = 15, and gap extension penalty (−E) = 8. The variations in samples were extracted by SAMtools software with following options: minimum mapping quality for SNPs (−Q) = 30, minimum mapping quality for gaps (−q) = 15, minimum read depth (−d) = 3, maximum read depth (−D) = 89, min Indel score for nearby SNP filtering (−G) = 30, SNP within INT bp around a gap to be filtered (−w) = 15, and window size for filtering dense SNPs (−W) = 15 [13]. For comparison of SNP calling sensitivity, we tested different pipelines for read mapping using the Bowtie2 aligner with default parameters.

### Orthologs retrieval between pepper and Arabidopsis genes

To take advantage of a published transcriptome analysis comparing YCM334 and Taean and performed using the *Arabidopsis* gene chip [17], we attempted to identify pepper genes orthologous to those *Arabidopsis* gene IDs previously identified as DEGs in this study. For this analysis we used the PLAZA 3.0 dicot database [22]. However, because this database does not currently cover the pepper genome, we first retrieved tomato gene IDs directly orthologous to the *Arabidopsis* gene IDs. Top pepper genes closely matching these tomato genes were then identified by BLASTP protein sequence alignment and were regarded as orthologs in the pepper gene model.

## Additional files

Additional file 1:
**Table S1**. Summary of raw read quality control. **Table S2**. Summary of SNP identification from current and previous researches. **Table S3**. List of pepper QTLs against various pathogens and the corresponding literatures. **Figure S1**. Comparison between NGS and Sanger sequencing result. Red colored bases are shared SNP calling from both Bowtie2 and BWA pipelines and green colored bases are additional SNPs only from Bowtie2 pipeline. Comparing with Sanger result, BWA pipeline showed no false positives and two false negative (10285038(T/A), 10285121(A/G)) while bowtie2 pipelines showed one false positive (10285103 (T/A)) and one false negative (10285038 (T/A)). **Figure S2**. Interproscan annotation of CA10g15480 and CA12G20430. (DOCX 442 kb)
Additional file 2: HRM primer list based on the polymorphic SNPs between YCM334 and Taean. (XLSX 63194 kb)
Additional file 3: CAPS primer set and restriction enzyme for detecting polymorphism on genic regions. (XLSX 6953 kb)
Additional file 4: Indel based CAPS primers. (XLSX 15 kb)
Additional file 5: The list of gene-associated SNPs with type, positions and sequence context. (XLSX 11974 kb)
Additional file 6: SNPs exerting the nonsynounymous protein changes on NBS-LRR genes. (XLSX 55 kb)
Additional file 7: CAPS genotyping results in 156 RIL population from a cross of YCM334 and Taean using selected SNP marker (CA04G03400 SNP1, Additional file 1: Figure S1) and BW resistance phenotype scored from 1 (most resistant) to 5 (most susceptible). (XLSX 13 kb)

## Abbreviations

BW: Bacterial wilt
CAPS: Cleaved amplified polymorphic sequences
CDS: Coding sequence
DEG: Differentially expressed genes
GBS: Genotype by sequencing
HRM: Resolution melting analysis
Indel: Insertion and deletion
NBS-LRR: Nucleotide-binding site-leucine-rich repeat
NGS: Next generation sequencing
Non-Syn: Nonsynonymous
QTL: Quantitative traits loci
RDA: Rural development administration
RIL: Recombinant inbred line
SNP: Single nucleotide polymorphism
