# Supplementary material for: Resequencing of Capsicum annuum parental lines (YCM334 and Taean) for the genetic analysis of bacterial wilt resistance
Source: BMC Plant Biol. 2016 Oct 28;16:235. doi: 10.1186/s12870-016-0931-0 (PMC5084322; doi:10.1186/s12870-016-0931-0)
Supplement: Additional file 1: — Table S1. Summary of raw read quality control. Table S2. Summary of SNP identification from current and previous researches. Table S3. List of pepper QTLs against various pathogens and the corresponding literatures. Figure S1. Comparison between NGS and Sanger sequencing result. Red colored bases are shared SNP calling from both Bowtie2 and BWA pipelines and green colored bases are additional SNPs only from Bowtie2 pipeline. Comparing with Sanger result, BWA pipeline showed no false positives and two false negative (10285038(T/A), 10285121(A/G)) while bowtie2 pipelines showed one false positive (10285103 (T/A)) and one false negative (10285038 (T/A)). Figure S2. Interproscan annotation of CA10g15480 and CA12G20430. (DOCX 442 kb) [file 12870_2016_931_MOESM1_ESM.docx]

| **Table S1**. Summary of raw read quality control | | | | |  |
| --- | --- | --- | --- | --- | --- |
|  |  | Total bases | | Sum | Coverage (X) |
|  |  | Read 1 | Read 2 |  |  |
| Raw reads | YCM334 | 18,438,326,488 | 18,438,326,488 | 36,876,652,976 | 10.60 |
|  | Taean | 17,975,211,895 | 17,975,211,895 | 35,950,423,790 | 10.33 |
| Trimmed | YCM334 | 15,516,739,072 | 15,533,262,034 | 31,050,001,106 | 8.92 |
|  | Taean | 14,862,671,830 | 14,813,423,857 | 29,676,095,687 | 8.53 |

| **Table S2.** Summary of SNP identification from current and previous researches | | | |
| --- | --- | --- | --- |
|  | Number of SNPs | | |
| Chromosomes | Known SNPs ^(1)^ | SNPs among four lines ^(2)^ | Newly identified SNPs |
| Pepper1.55ch01 | 1,911,460 | 2,111,058 | 199,598 |
| Pepper1.55ch02 | 1,057,458 | 1,234,872 | 177,414 |
| Pepper1.55ch03 | 1,191,993 | 1,360,049 | 168,056 |
| Pepper1.55ch04 | 791,533 | 908,852 | 117,319 |
| Pepper1.55ch05 | 1,214,868 | 1,492,777 | 277,909 |
| Pepper1.55ch06 | 1,317,374 | 1,572,843 | 255,469 |
| Pepper1.55ch07 | 1,429,671 | 1,608,250 | 178,579 |
| Pepper1.55ch08 | 345,857 | 459,868 | 114,011 |
| Pepper1.55ch09 | 2,935,773 | 3,404,767 | 468,994 |
| Pepper1.55ch10 | 2,134,798 | 2,444,767 | 309,969 |
| Pepper1.55ch11 | 2,300,681 | 2,640,630 | 339,949 |
| Pepper1.55ch12 | 923,461 | 1,064,358 | 140,897 |
| Total | 17,554,927 | 20,303,091 | 2,748,164 |
| (1) known SNPs from previous research (Kim et al. 2014) using Dempsey and Perennial cultivars | | | |
| (2) SNPs among four cultivars Dempsey, Perennial, YCM334, and Taean | | | |

| Table S3. List of pepper QTLs against various pathogens and the corresponding literatures | | | | | | |
| --- | --- | --- | --- | --- | --- | --- |
| Phenotype | Marker name | Chr | Position | PrimerF | PrimerR | Reference |
| Resistance to *Phytophthora capsici* | CAMS420 | 5 | 32037641-32037840 | cagcgttctatcgtctcaaatg | ttgacaaaccagaaattgatcg | Minamiyama et al. 2007 |
|  | CAMS612 | 8 | 48317076-48317285 | tccaccatgaatcgaagaca | agtcgcatcctgtccaaagt | Minamiyama et al. 2007 |
|  | C2_At2g01770 | 5 | 38128630-38129027 | ACCATGTATGAAAGGAGTTGTACCTCG | AATTTACAGCAACTTGCATATGGAGA | Mallard et al. 2013 |
|  | CAMS072 | 5 | 1888412-1888554 | cccgcgaaatcaaggtaat | aaagctattgctactgggttcg | Mallard et al. 2013, Truong et al. 2012* |
|  | CA524065 | 5 | 214215595-214215833 | TCTCTCTCTACATCTCTCCGTTG | TGTCGTTCGTCGACGTACTC | Truong et al. 2012 |
| Resistantce to anthracnose  (*Colletotrichum acutatum*) | 244687-AN12 | 12 | 226881424-226881478 | TCTCCTCAACACTTTACTATGTTCG | CACTAAATAGTGTCCCCTCACC | Korea Patent 10-1266193 |
|  | 570374-AN12 | 12 | 217851050-217851143 | GCTCAGAGATTTGGTTGAATTAGTC | ATTCTCGATTGAATTTATGGAAGT | Korea Patent 10-1266193 |
|  | 38820-AN4 | 4 | 205238553-205238629 | CATCCAGTTAGTTCTGTTATATTTTCA | CGAGTTACAATTATGCAGGAACAC | Korea Patent 10-1266193 |
|  | 284900-AN4 | 4 | 210106382-210106448 | ATTTTAAATGGAAACTGGCGAAC | ACAAACAAGTGCACTAGGAACAAG | Korea Patent 10-1266193 |
|  | Anth12 | 1 | 154226189-154226283 | AATATTTTTCACCTATAAAGCACA | ACACGTGTAAGCTCTAATTCAA | Korea Patent 10-1266193 |
| Resistance to *Ralstonia solanacearum* | CAMS-451 | 8 | 124766791-124767001 | tgcattggtgggctaacata | gctcttgacacaaccccaat | {Mimura, 2009 #2323} |
| * Truong et al. showed the AFLP markers between HpmsE015 and CA524065 are highly related with disease resistance; however, HpmsE015 was failed to map on reference genome. Instead, we used proximal marker, CAMS072, reported from Mallard et al. 2013 | | | | | | |


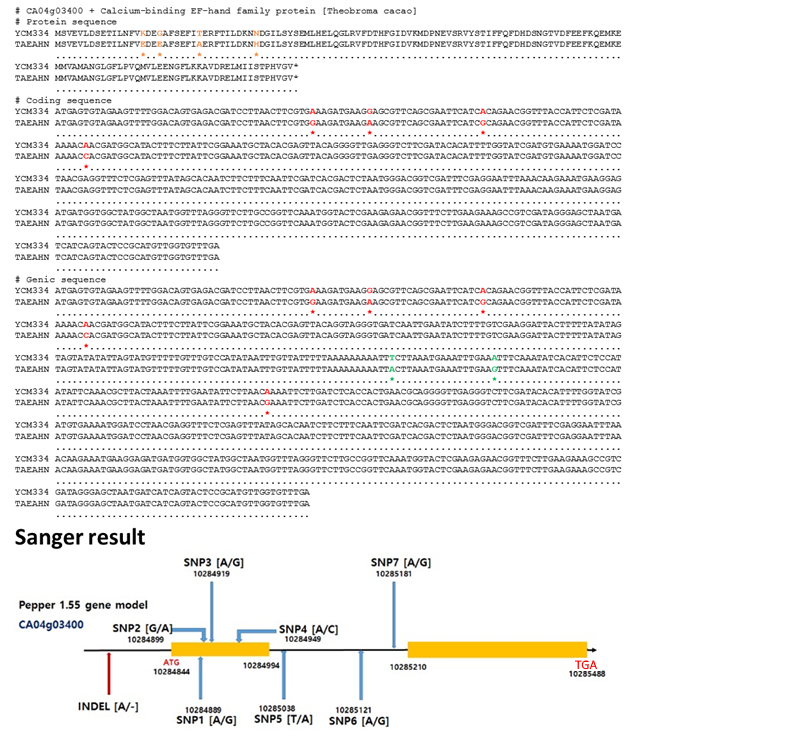


**Supplementary Figure 1.** Comparison between NGS and Sanger sequencing result. Red colored bases are shared SNP calling from both Bowtie2 and BWA pipelines and green colored bases are additional SNPs only from Bowtie2 pipeline. Comparing with Sanger result, BWA pipeline showed no false positives and two false negative (10285038(T/A), 10285121(A/G)) while bowtie2 pipelines showed one false positive (10285103 (T/A)) and one false negative (10285038 (T/A)).


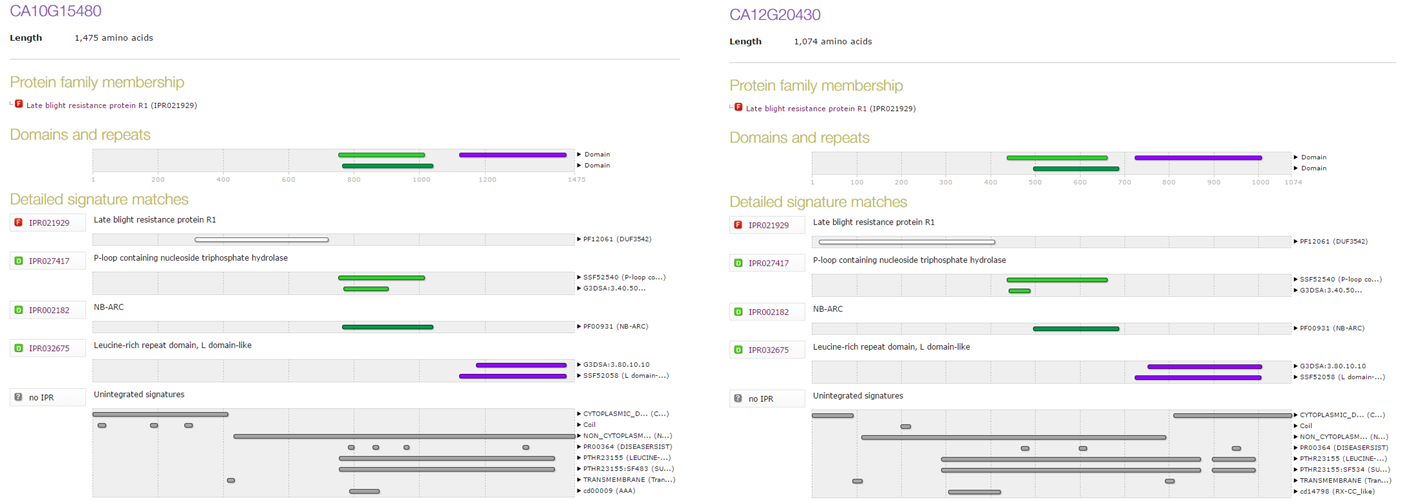


**Supplementary Figure 2.** Interproscan annotation of CA10g15480 and CA12G20430
